# Recurrent paralysis, aseptic meningitis: Unusual discovery circumstance of a ruptured epidermoid cyst

**DOI:** 10.1016/j.radcr.2025.04.092

**Published:** 2025-05-27

**Authors:** Mohamed Soufiane Kaddouri, Firdaous Touarsa, Badr Kabila, Mohamed Jiddane

**Affiliations:** a Hôpital des Spécialités, Neuro-radiology, Rabat-Salé-Kénitra, Rabat, Morocco; b Mohammed V Souissi University, Neuro-radiology, Rabat-Salé-Kénitra, Rabat, Morocco

**Keywords:** Aseptic meningitis, Epidermoid cyst, MRI

## Abstract

Intracranial epidermoid cysts are benign tumors of congenital origin. They result from the aberrant inclusion of ectodermal elements during neural tube closure between the third and fifth weeks of embryonic development. These tumors typically present with local mass effect. We report an exceptional case of a ruptured intracranial epidermoid cyst, revealed by recurrent paralysis and aseptic meningitis.

## Introduction

Intracranial epidermoid cysts are slow-growing, benign congenital tumors [1]. They arise from ectodermal elements trapped during neural tube closure and are commonly found in the cerebellopontine angle [2]. These cysts account for approximately 0.2% to 1.8% of all intracranial tumors and are often asymptomatic for extended periods due to their slow growth [3]. When symptoms do appear, they are typically related to mass effect on adjacent neurovascular structures, leading to cranial nerve deficits, hydrocephalus, or seizures depending on the cyst's location [4].

Although most epidermoid cysts present with symptoms related to compression, spontaneous rupture is a rare and often underrecognized complication. The rupture of an epidermoid cyst results in the dissemination of its keratin and cholesterol-rich content into the subarachnoid space, triggering an inflammatory response known as chemical or aseptic meningitis [5]. This inflammatory process can lead to severe neurological symptoms, including headaches, meningeal irritation, and even cerebrovascular complications such as vasospasm or infarction [6]. Due to its rarity and nonspecific presentation, ruptured epidermoid cysts can pose a diagnostic challenge and require high clinical suspicion and appropriate imaging for accurate identification.

**Fig. 1:**
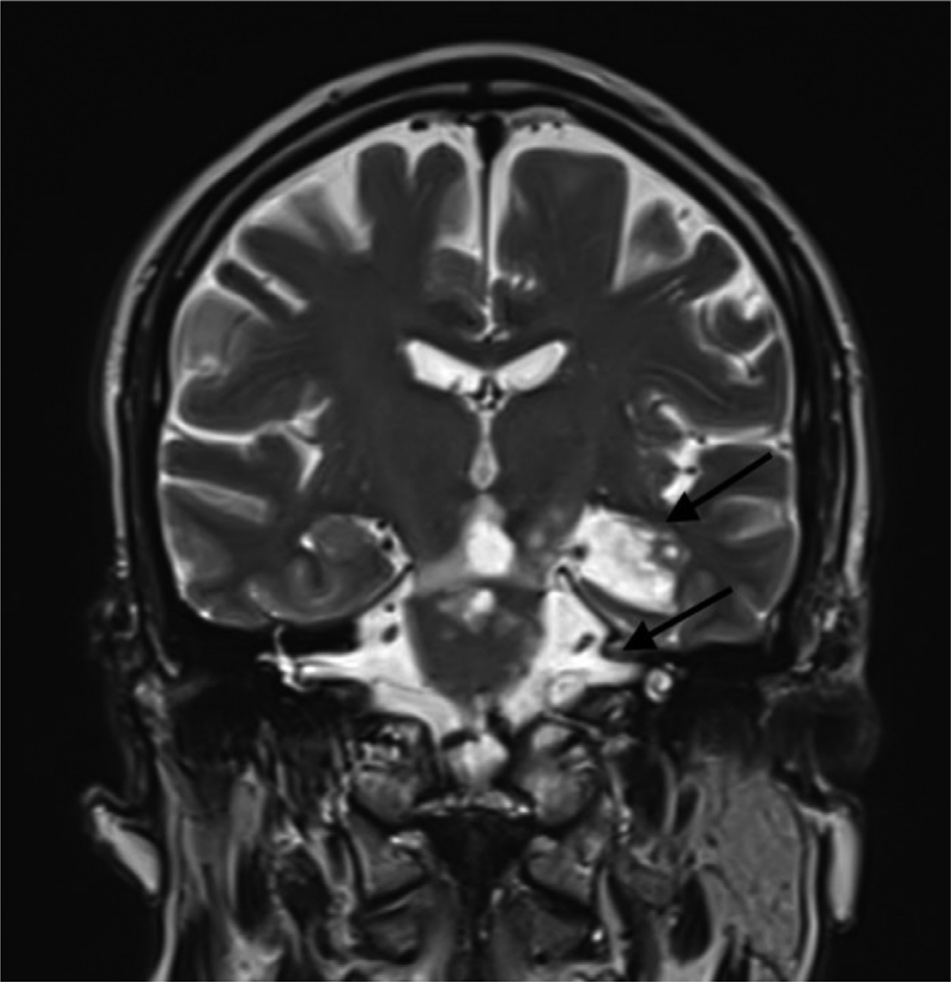
Coronal T2 weighted sequence showing an enlargement of the left choroidal fissure (Arrow) and para-pontic and bulbar cisterna (Arrow), filling with epidermoid cyst hyperintense in T2.

**Fig. 2:**
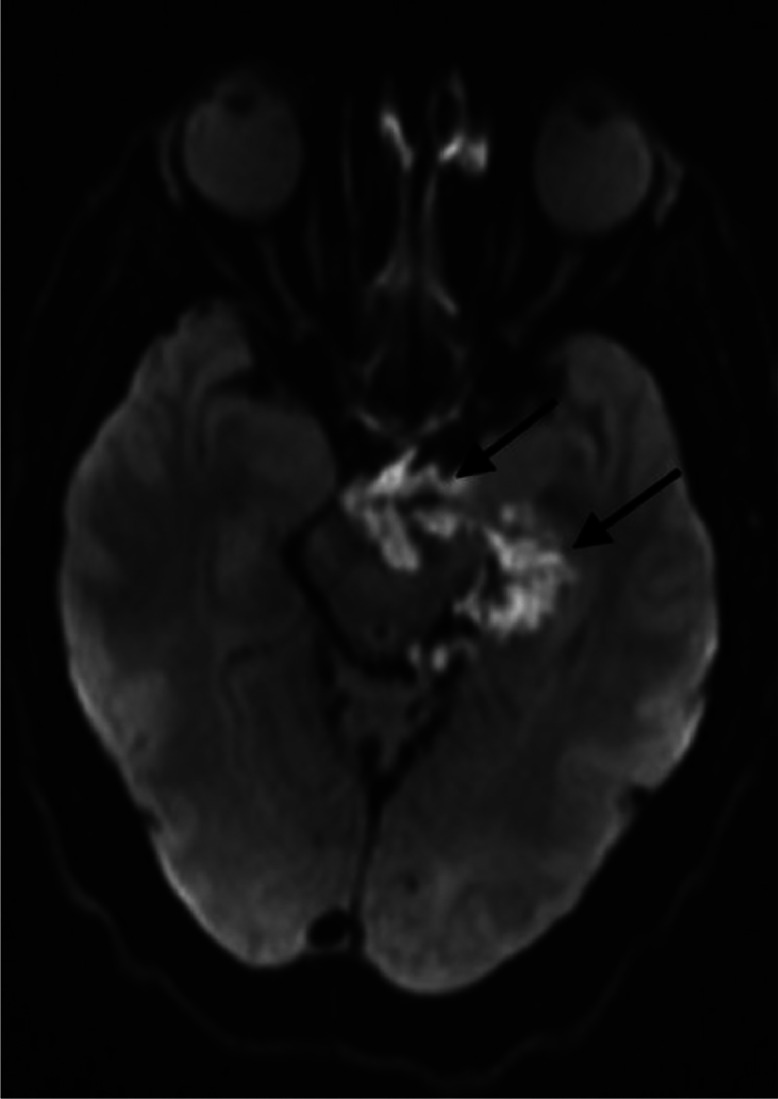
Diffusion-weighted sequence showing a ruptured epidermoid cyst of the left choroidal fissure (arrows).

**Fig. 3:**
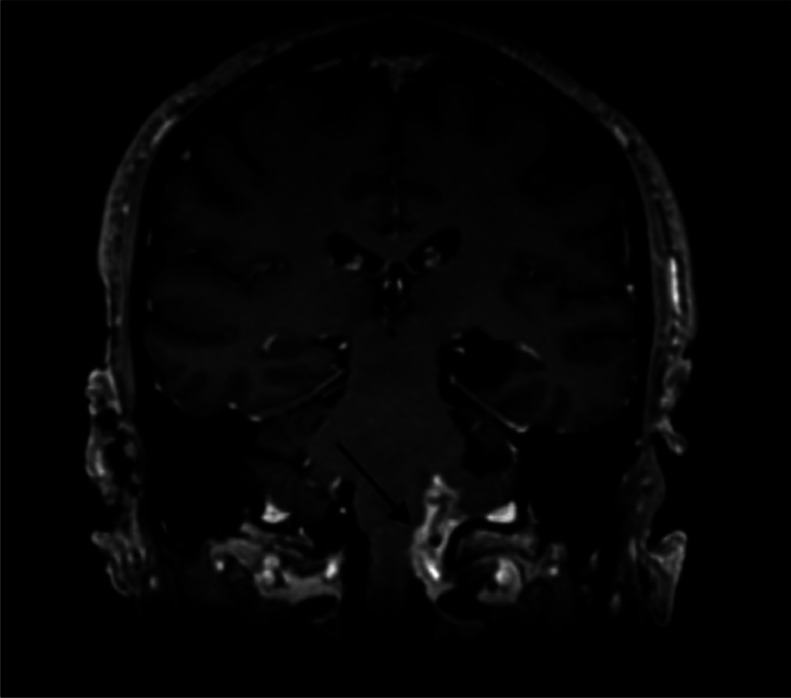
Coronal T1 weighted sequence with gadolinium-based contrast agent injection revealed leptomeningeal enhancement in the left lateral bulbar (arrow).

**Fig. 4:**
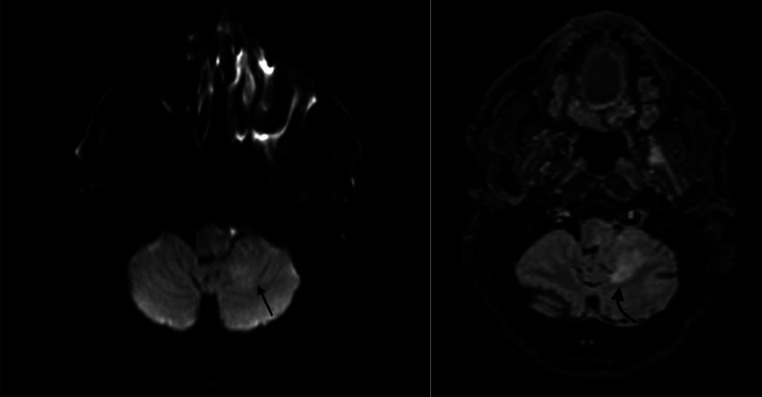
Axial FLAIR weighted sequence/ Diffusion-weighted sequence revealed ischemic signal anomaly at the left bulbo-cerebellar level (Curved arrow), hyperintense in FLAIR (A), showing mild diffusion hyperintensity, with high apparent diffusion coefficient (ADC), well involving the left vagus et glossopharyngeal nerves nucleus (straight arrow), consisting in venous infarcts (B).

MRI is the imaging modality of choice for diagnosing epidermoid cysts and their complications. Diffusion-weighted imaging (DWI) is particularly useful in distinguishing these lesions from arachnoid cysts, as epidermoid cysts exhibit diffusion restriction due to their solid nature. Prompt diagnosis and management are crucial to prevent complications and optimize patient outcomes (Fig. 1, Fig. 2, Fig. 3, Fig. 4).

Here, we describe a case of a 30-year-old patient presenting with recurrent paralysis as the initial manifestation of a ruptured epidermoid cyst, emphasizing the importance of early recognition and intervention in such cases.

## Case presentation

A 30-year-old male with no previous medical or surgical history and no family history of neurological disorders presented with sudden onset of left-sided vagus nerve palsy, recurrent laryngeal nerve palsy, and palatal dysfunction, leading to dysphonia and mild dysphagia. The patient also reported intermittent headaches and dizziness over the preceding few weeks. Neurological examination revealed hoarseness of voice, decreased left palatal elevation, an absent gag reflex on the left side, mild left-sided tongue deviation, mild dysmetria on the left side, but no limb weakness or sensory deficits. Routine blood tests showed a White Blood Cell (WBC) count of 7200/mm³ (Normal: 4000-11,000/mm³), C-Reactive Protein (CRP) of 1.2 mg/L (Normal: <5 mg/L), and an Erythrocyte Sedimentation Rate (ESR) of 10 mm/h (Normal: <20 mm/h), with a normal coagulation profile. Cerebrospinal Fluid (CSF) analysis revealed WBC count of 75/mm³ (predominantly lymphocytes) (Normal: <5/mm³), protein of 60 mg/dL (Normal: 15-45 mg/dL), glucose of 55 mg/dL (Normal: 40-70 mg/dL), and negative cultures for bacteria, fungi, and viruses, with no malignant cells.

MRI showed a ruptured epidermoid cyst in the left choroidal fissure, hypointense on T1, hyperintense on T2 and FLAIR, with diffusion restriction. The left lateral-bulbar subarachnoid spaces were filled with similar signal characteristics, with leptomeningeal enhancement in the left lateral bulbar and cerebellar regions on postcontrast imaging. A left lateral bulbar and cerebellar venous infarction involving the vagus and glossopharyngeal nerve nuclei was also noted.

Management included corticosteroids (dexamethasone 10 mg IV followed by tapering doses) to reduce inflammation and edema [4], empirical antibiotic therapy (ceftriaxone and vancomycin) until bacterial meningitis was ruled out, and supportive care including IV hydration, analgesia, and speech therapy. The patient did not undergo surgical resection.

The patient showed gradual improvement in neurological symptoms over 4 weeks, with significant recovery in voice and swallowing function.

## Discussion

Epidermoid cysts are benign congenital lesions arising from residual ectodermal epithelium. The majority of epidermoid cysts are located in the cerebellopontine angular cistern, followed by the prepontine, suprasellar, and parasellar regions [2]. On gross pathology, epidermoid cysts are well-circumscribed lesions with a nodular surface and a shiny “pearlescent” appearance, from which the term “pearlescent tumor” is derived. Sometimes calcification occurs. They are lined with stratified squamous epithelium and cilia, which gradually shed and keratin degrade, causing the lesions to grow over time. These cysts are filled with soft, waxy, or scaly material rich in debris, keratin, water, and cholesterol crystals [3].

Intracranial epidermoid cysts are discovered in a variety of circumstances, most often involving extrinsic compression of nearby nerve structures. Rupture is an exceptional complication [6]. MRI with gadolinium injection is the imaging modality of choice for the diagnosis of intracranial epidermoid cysts and its complications. The typical imaging finding is a well-circumscribed lobulated mass that is hypodense on CT scan, hypointense on T1-weighted MRI, and hyperintense on T2-weighted and DWI [5]. High signal on DWI corresponds to low signal on apparent diffusion coefficient (ADC) maps. This diffusion restriction indicates the solid nature of the lesion and limited hydrogen proton diffusion, distinguishing epidermoid cysts from other cerebrospinal fluid-filled cysts, such as arachnoid cysts. The diffusion restriction of epidermoid cysts may be explained by the precise spatial organization of the lesions and their tendency to grow in multiple epithelial layers [4].

Rupture is a rare complication. Radiological diagnosis is based on demonstrating the epidermoid cyst's contents in the supratentorial and subtentorial cisternal system, depending on its initial location [6]. Rupture can cause episodic aseptic meningitis by releasing cyst contents into the subarachnoid space, which results in chemical irritation. On imaging, aseptic meningitis appears as a pachymeningeal or leptomeningeal thickening, enhancing after gadolinium injection [3].

In our patient, the aseptic meningitis was complicated by a left lateral bulbar venous infarction involving the left vagus and glossopharyngeal nerve nuclei, explaining the patient’s symptoms, along with left cerebellar venous infarction. Cerebral infarction secondary to meningitis can be caused by increased cerebral vasospasm, vasculitis, impairment of the coagulation cascade, and systemic inflammatory responses [2]. In this case, the lateral bulbar and left cerebellar infarction likely resulted from venous congestion due to inadequate venous drainage caused by the left lateral bulbar meningitis.

Treatment is based on the complete surgical removal of the primary tumor capsule and intracystic contents without causing damage to the surrounding neurovascular structures [5]. Long-term follow-up is crucial to monitor for recurrence.

## Conclusion

Intracranial epidermoid tumors are rare, slow-growing tumors that can present with rupture and aseptic meningitis, leading to ischemic neurovascular complications. Early diagnosis with MRI, combined with appropriate surgical intervention, results in favorable outcomes. This case highlights an unusual presentation with recurrent paralysis and emphasizes the importance of recognizing rupture as a potential complication.

## Ethics approval

Our institution does not require ethical approval for reporting individual cases or case series.

## Patient consent

Written informed consent was obtained from the patient(s) for their anonymized information to be published in this article.
